# Promoting HIV care continuum outcomes among people who use drugs and alcohol: a systematic review of randomized trials evaluating behavioral HIV care interventions published from 2011 to 2023

**DOI:** 10.1186/s12889-023-17113-5

**Published:** 2023-11-07

**Authors:** Eileen V. Pitpitan, John Mark Wiginton, Raul Bejarano-Romero, Dania Abu Baker

**Affiliations:** 1 School of Social Work, San Diego State University, 5500 Campanile Drive, San Diego, CA 92182-4119 USA; 2 Division of Infectious Diseases and Global Public Health, School of Medicine, University of California San Diego, 9500 Gilman Drive, La Jolla, CA 92093 USA; 3 San Diego State University, University of California-San Diego Joint Doctoral Program in Interdisciplinary Research on Substance Use, San Diego, CA USA

**Keywords:** HIV care continuum, People living with HIV (PLHIV), Linkage to care, ART uptake, ART adherence, Retention in care, Viral suppression, Drug use, Substance use, Systematic review

## Abstract

**Background:**

Substance use remains a robust predictor of HIV infection and a serious impediment to HIV care continuum progression for people living with HIV. The primary research question of this systematic review is focused on understanding the extent to which behavioral HIV care interventions have been efficacious in helping people who live with HIV and who use substances along the HIV care continuum.

**Methods:**

Using PubMed and ProQuest databases, we performed a systematic review of randomized trials of behavioral HIV care continuum interventions among people who use substances published from 2011 to August 2023, since the beginning of the treatment-as-prevention era.

**Results:**

We identified 11 studies (total participants: N = 5635), ten intentionally targeting substance-using populations. Four studies involved samples using ≥ 1 substance (e.g., alcohol, opioids, stimulants, marijuana); four involved injection drug use; one involved methamphetamine use; and one involved alcohol use. One study targeted a population with incidental substance use (i.e., alcohol, injection drug use, non-injection drug use reported in most participants). Each study defined one or more HIV care outcomes of interest. Viral suppression was an outcome targeted in 9/11 studies, followed by uptake of antiretroviral therapy (ART; 7/11), ART adherence (6/11), retention in care (5/11), and linkage to care (3/11). While most (nine) of the studies found significant effects on at least one HIV care outcome, findings were mostly mixed. Mediated (2/11) and moderated (2/11) effects were minimally examined.

**Conclusions:**

The results from this systematic review demonstrate mixed findings concerning the efficacy of previous HIV care interventions to improve HIV care continuum outcomes among people who use substances. However, heterogeneity of study components (e.g., diversity of substances used/assessed, self-report vs. objective measures, attrition) prevent broad deductions or conclusions about the amenability of specific substance-using populations to HIV care intervention. More coordinated, comprehensive, and targeted efforts are needed to promote and disentangle intervention effects on HIV care continuum outcomes among substance-using populations.

## Background

Substance use remains a robust predictor of HIV infection across resource-diverse contexts and settings [1–4]. A recent systematic review found that injecting drugs, smoking crack cocaine, and binge drinking predicted HIV infection among adults in high-income countries [4]. Separate reviews similarly report that injecting drugs and using stimulants (e.g., methamphetamines) contributed to HIV burden in lower-resource environments [5]. Pathways linking substance use to HIV infection include direct routes, such as needle sharing among people who inject drugs, and indirect routes, such as behavioral disinhibition (e.g., through heavy alcohol or stimulant use) in the form of condomless sex [2, 5–9].

Among people living with HIV (PLHIV), substance use has also been shown to impede progress at multiple stages of the HIV care continuum, from late HIV diagnosis to treatment failure [2], accelerating HIV disease progression [10]. For example, engaging in heavy alcohol use – harmful or hazardous alcohol use, binge drinking, or levels of drinking consistent with those seen in alcohol use disorders – has been shown to hamper uptake of antiretroviral therapy (ART), decrease ART adherence and CD4 cell count, increase viral load, and accelerate HIV disease symptom onset [3, 6, 11, 12]. Likewise, a qualitative study involving people engaged in injection and non-injection drug use (marijuana, heroin, cocaine, methamphetamines) found that substance use prevented or delayed HIV testing and linkage to and retention in care, and derailed ART adherence [13]. Other research has demonstrated that injection drug use and stimulant use negatively affect retention in care and ART adherence (even resulting in discontinuation) and increase viral load [14–18]. Notably, substance use itself (stimulant use in particular) may facilitate viral replication, thereby leading to higher viral load, regardless of ART adherence [10, 19].

Given the substantial role that substance use plays in HIV care continuum outcomes, research is needed to inform the design and implementation of behavioral HIV care interventions to promote HIV treatment outcomes (e.g., engagement in care, ART adherence) among people who use alcohol or drugs. A necessary step in these efforts is to understand the extent to which behavioral HIV care interventions have been efficacious in helping people who use substances progress across the HIV care continuum. It is also imperative to note instances in which key factors, informed by behavior change theory [20, 21], have been examined as potential mediators (e.g., HIV treatment self-efficacy or ART adherence mediating the path to viral suppression) or moderators (e.g., by substance used, mental health, gender, age) in analyses of behavioral HIV care intervention outcomes, which would increase understanding of the mechanisms through which these interventions have operated to impact outcomes, as well as highlight which subgroups of individuals have been more or less affected by interventions. Recent research has systematically reviewed the literature on substance use treatment interventions, specifically medications for opioid use disorder, and their effect on infectious disease outcomes, including HIV care outcomes [22]. However, to date, no systematic review research has been conducted specifically on behavioral HIV care interventions in affecting HIV care outcomes among people who use substances. Thus, we sought to fill this gap in the literature with the current systematic review.

The objectives of this paper were first to review published literature from January 2011-August 2023 to examine the extent to which behavioral HIV prevention and HIV care continuum interventions have been efficacious for people who use drugs and/or alcohol. We selected 2011 as the starting point because this was the beginning of the treatment as prevention (TasP) era when test-and-treat strategies were beginning to be understood and implemented [23–25]. Another goal of this review was to explore the extent to which mediators and moderators have been tested as part of the outcome analyses for these interventions.

## Methods

### Eligibility criteria

A study was eligible for inclusion if it (1) focused on people at risk for or living with HIV; (2) evaluated the efficacy of a behavioral intervention; (3) included people who reported active or recent drug or alcohol use (≥ 50% of the sample), or intentionally targeted a substance-using population; (4) examined HIV prevention or HIV care continuum outcomes (any of the following: uptake of HIV pre-exposure prophylaxis [PrEP]; PrEP adherence; linkage to HIV care; retention in HIV care; ART uptake, use, or adherence; HIV viral load or viral suppression; or immune function [e.g., CD4 count]); (5) used a randomized controlled trial design; and (6) sampled ≥ 200 participants. The latter two criteria were added toward the end of article selection to restrict the review to studies using the gold-standard design for evaluating intervention efficacy, to eliminate smaller pilot trials, and because moderation/mediation analyses require dividing the sample across different subgroups, which is difficult with small sample sizes. We did not include systematic literature reviews. Altogether, the eligibility criteria were selected to inform the development of a secondary analysis study led by the first author focused on examining mediators and moderators in randomized trials evaluating behavioral HIV care interventions for people who use substances (NIH R01DA058311).

### Literature search

#### Search strategy

**Fig. 1:**
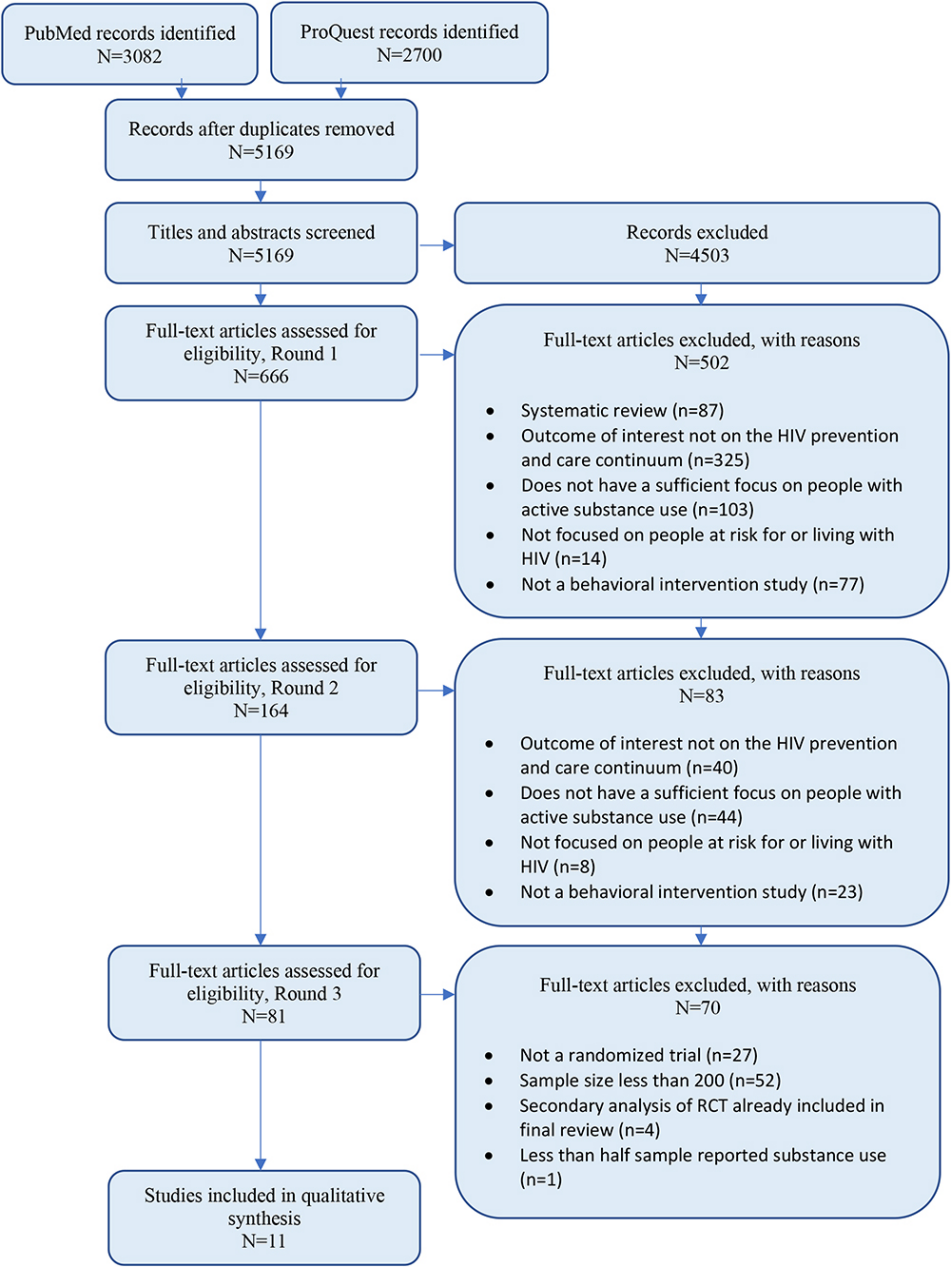
PRISMA flow diagram

In September 2021, we conducted electronic searches of articles indexed in PubMed and ProQuest between 2011 through September 2021; we repeated our search in August 2023, extending the end point of our previous range of review to August 2023. Our PubMed search using the terms (HIV intervention) AND (prep OR pre-exposure OR treatment OR care OR adherence OR viral) AND (drug OR substance) yielded 3082 articles published since 2011 (Fig. 1). Our ProQuest search using the terms (HIV) AND (intervention) AND (treatment OR care OR adherence OR ART adherence OR viral OR pre-exposure OR PrEP) AND (drug OR substance OR misuse OR dependence OR addict*) yielded 2700 articles published since 2011. We exported all 5782 records to DistillerSR (Evidence Partners, Ottawa, Canada), an online systematic review automation tool. After removing duplicates, we used the same software to screen the remaining 5169 articles based on the criteria mentioned above. During our review process, we identified 19 articles that were systematic reviews about HIV and substance use [26–44]. We examined these articles and their reference lists and found no additional studies that met the inclusion criteria.

#### Selection process

Three reviewers (EVP, RB, DAB) – who were not blind to the authors, funding, or any study characteristic – independently screened a small subset of the 5169 articles and discussed disagreements to promote rater reliability. Reviewers then independently screened abstracts from the same subset of 1250 (25%) articles identified in the initial search to determine interrater reliability. Reviewers were instructed to include “HIV intervention studies” and exclude editorials, opinion papers, and qualitative studies. Reviewers exercised liberal judgment for the initial inclusion of potentially relevant articles for further review and met weekly to discuss and resolve disagreements. Interrater reliability was high (kappa = 0.98).

Two reviewers independently screened the remaining abstracts, continuing with the liberal approach, which resulted in excluding 4503 articles and retaining 666 articles for full-text screening. For this process, two reviewers independently screened the full-text of the remaining articles and adjudicated eligibility criteria to determine inclusion in the final sample. Reviewers considered the following questions to determine eligibility: (1) Is this a systematic review? (2) Is this study focused on people at risk for or living with HIV? (3) Does this paper focus on people with active substance use, either as described by the authors or determined by the reviewer from the sample description? (4) Is this an intervention study? (5) Is the outcome a variable in the HIV prevention and/or care continuum? Reviewers could indicate “yes” or “no” in response to these questions, and could also indicate “unsure” for questions 2 through 5. Any response other than “no” (exclusion) for a given article resulted in the article being included in a second round of review to assess eligibility.

After this round of full-text screening, 502 articles were excluded, and 164 were retained for further review. A second round of review resulted in 83 articles being excluded, leaving 81 for the final phase of review. At this third round of the article selection phase, we added the two additional inclusion criteria described previously: randomized controlled trial design and a sample size of 200 + participants. After this stage, 65 articles were excluded. An additional five papers were later excluded – four because they were found to be secondary analyses of a study already included in the final review, and one because less than half of the sample reported substance use, leaving eleven articles for the present review. All reviewers agreed upon the studies included in the final sample.

#### Data collection process

Two reviewers examined the articles and extracted data from multiple domains: study authors; year published; recruitment time frame; population targeted (as described by original authors); city/region where the study was conducted; sample size; mean or median age of the sample; name of intervention being tested (if available); whether and how the intervention was described socio-ecologically (individual, community, structural, multilevel); all intervention strategies (active ingredients); whether the article described the intervention as theory-informed (and what theories informed the design); intervention format, length, and delivery; primary outcome(s) of interest; secondary outcome(s) of interest; proportion successful in comparison group(s) (as reported); proportion successful in intervention group (as reported); p-value and other relevant statistics regarding main intervention effects on the primary outcome (with interpretation); whether the analytic approach was theory-informed; whether a mediation and/or moderation analysis was reported (if so, the analytic approach and variables tested); and missingness assumptions. The authors created a table displaying all extracted data for easy comparison and examination. All study authors confirmed the accuracy of the data extraction results.

#### Risk of bias assessment

Risk of bias was independently assessed by two reviewers who reviewed the methodological quality of the studies included using the Cochrane risk-of-bias tool (RoB2) for the RCT design [45]. The Cochrane RoB2 includes questions about randomization, deviations from intended interventions, missing outcome data, measurement of the outcome, selection of the reported result, and overall risk-of-bias [45]. Each domain was assessed as either “high,” “low,” or “some concerns” about bias per study for each domain, and for the overall risk-of-bias judgment. Any disagreements between the two reviewers were discussed and the reviewers reached consensus around final judgments.

## Results

Eleven studies met the final inclusion criteria (total participants: N = 5635; Table 1). All identified interventions sought to promote progress at one or multiple stages of the HIV care continuum, and several sought to address additional outcomes (e.g., reduced drug use, reduced condomless sex). No interventions to promote PrEP continuum outcomes were identified.

**Table 1:** Description of studies included in the review

Author, publication year, design, location, recruitment period	Study population, sample size, key demographics	Relevant substance-use characteristics	Intervention, experimental arms, follow-up visits from baseline	Targeted HIV care continuum (HCC) and other outcomes	Attrition	Overall Efficacy on HIV Care Continuum Outcomes	Mediation or moderation	
Attonito et al., 2019; two-arm RCT; Miami, Florida, USA; January 2009-November 2012	PLHIV recruited from substance use treatment facilities and HIV care and service organizations; N = 243	Screened positive for harmful/ hazardous drinking via AUDIT; 46%, prior treatment for alcohol use; use of other substances not assessed	Holistic Health Recovery Program-Adapted (HHRP-A); HHRP vs Health Promotion Comparison (HPC); 3 and 6 months	HCC: ART-adherence (self-report, 7-day recall), HIV service utilization (self-report), viral load (self-report)	208/243 completed baseline; 183 and 160 provided data at 3 and 6 mo, respectively	Significant intervention effects at study endpoint on ART adherence and viral suppression, no effect on retention (service utilization)	No	
Satre et al., 2019; three-arm RCT; San Francisco, California, USA; April 2013-May 2015	PLHIV recruited from HIV primary care clinic; N = 614	Past-year unhealthy alcohol use (self-report of at least 1 instance of ≥ 3 drinks/day for women and ≥ 4 drinks/day for men in past year); 57% at high risk for alcohol use problems; 25% met DSM-IV criteria for alcohol dependence	Unnamed; Motivational interviewing (MI) vs Emailed feedback vs usual care; 6 and 12 months	HCC: ART adherence (30-day recall), viral suppression (clinical chart review) Other: reduced substance use in past 30 days (self-report), importance of reducing alcohol use (self-report)	614/614 included in primary analysis; 582 and 583 provided data at 6 and 12 mo, respectively	No significant intervention effects at study mid or endpoints on ART adherence or HIV viral control	No	
Samet et al., 2019; two-arm RCT; St. Petersburg, Russia; July 2012-May 2014	PLHIV who inject drugs not on ART hospitalized at City Addiction Hospital; N = 349	History of injection drug use and admitted into a narcology hospital	Linking Infectious and Narcology Care (LINC), a peer-led strengths-based case management intervention; LINC vs standard of care; 6 and 12 months	HCC: linkage to HIV care, CD4 cell count at 12 months, retention in HIV care within 12 months, appropriate engagement in HIV care (ART uptake or a second CD4 cell count if CD4 > 350 cells/µL) (all obtained via clinical chart review) Other: self-reported hospitalizations at 12 months	349/349 included in primary analysis; 249 and 244 provided data at 6 and 12 mo, respectively	Significant intervention effect at study midpoint on linkage to HIV care; no effects on ART uptake, retention, or CD4 at endpoint	No	
Samet et al., 2023; two-arm RCT; St. Petersburg, Russia; September 2018-December 2020	PLHIV who inject drugs not on ART hospitalized at City Addiction Hospital; N = 225	History of injection drug use and admitted into a narcology hospital	Linking Infectious and Narcology Care (LINC-II), a multicomponent, peer-led strengths-based case management, rapid access to ART, and receipt of naltrexone intervention; LINC-II vs standard of care; 6 and 12 months	HCC: viral suppression, ART initiation 28 days post randomization, retention in care, CD4 cell count Other: opioid abstinence	137/225 included in primary outcome analysis; 165/225 provided data at 12 mo	Significant intervention effect at study endpoint on viral suppression, ART uptake, and retention in care; no effect on CD4	No	
Myers et al., 2018; two-arm RCT; San Francisco, California, USA; 2010–2013	Substance-using PLHIV recently arrested and released from San Francisco County Jail; N = 270	All reported prior or current substance use; alcohol use, 94% used in 30 days prior to jail, 50% used weekly; drug use, 94% used in 30 days prior to jail, 76% used weekly; methamphetamines, 63% in 30 days prior to jail, 40% more than once/week; crack-cocaine, 57% used in 30 days prior to jail, 37% used more than once/week; heroin, 30% used in 30 days prior to jail, 13% used more than once/week; 33% met criteria for alcohol abuse via AUDIT; 85% met criteria for substance abuse, 8% severe substance abuse (DAST)	The NAV intervention (navigation-enhanced HIV case management) vs treatment as usual; 2, 6, and 12 months	HCC: linkage to HIV care, retention in HIV care, viral load, viral suppression (all abstracted from jail- and city-based laboratory databases) Other: reduction in risky sex (unspecified), reduction in risky drug use (weekly self-report, urinalysis)	270/276 included in primary analysis; 215, 203, and 221 provided data at 2, 6, and 12 mo, respectively	Significant intervention effects at study endpoint on linkage to and retention in care, no effect on viral load	No	
Metsch et al., 2016; three-arm RCT; 11 urban sites in the United States; July 2012-January 2014	Hospitalized PLHIV with elevated viral load; N = 801	Reported or documented opioid, stimulant, or heavy alcohol use (AUDIT-C) in past year; at baseline, 59% harmful/ hazardous alcohol use (AUDIT-C); 97%, drug use, including 69%, stimulants; 22%, opioids; 18%, injection drug use; 33%, severe substance use (DAST)	Patient navigation with/without financial incentives vs treatment as usual; 6 mo, 12 mo	HCC: HIV viral suppression at 6 and 12 mo (laboratory testing, unspecified), outpatient care with HIV specialist and being prescribed HIV medication (assessment method unspecified), HIV medication adherence at 6 and 12 mo (self-report, 30-day recall) Other: attending substance use treatment at 6 and 12 mo, level of substance use (urinalysis, self-report [12-month, 1-month recall]; injection drug use via GAIN), substance use severity (self-report via DAST-10, AUDIT-C)	774/801 included in primary analysis; 761 and 752 provided data at 6 and 12 mo, respectively	Significant intervention effect on viral suppression, HIV care visits (retention), and ART use at study midpoint; no effect on ART adherence; no effects at study endpoint	Yes, mediation, but results not reported in this paper Yes, moderation by site, baseline viral suppression, stimulant use, race, ethnicity, sex	
Miller et al., 2019; two-arm RCT; Kyiv, Ukraine; Thai Nguyen, Vietnam; Jakarta, Indonesia; February 2015-June 2016	PLHIV who inject drug with elevated viral load; N = 1308	Active injection drug users: self-reported ≥ 2 times/wk for past 3 mo and display most recent injection site; at 8 mo: self-reported ≥ 12 times for past 3 mo, ≥ 6 times for past mo, confirmed PWID by site staff	Harm reduction, systems navigation, psychosocial counseling, ART at any CD4 cell count vs Harm reduction/ standard of care; 12 mo	HCC: Uptake/use of ART (self-report), viral suppression (laboratory testing, unspecified) Other: medication-assisted drug treatment (self-report) mortality (unspecified; investigation by local team, deaths categorized by physicians)	908/1308 included in primary analysis, with 400 lost to follow-up	Significant intervention effects at study mid and endpoint on ART use and viral suppression	No	
Parsons et al., 2018; two-arm RCT; New York City, New York, USA; August 2008-December 2011	MSM LHIV with suboptimal ART-adherence who use methamphetamines; N = 210	Participants had used methamphetamines an average of six days in the past month	Motivational interviewing plus cognitive behavioral skills vs attention-matched education control; 3, 6, 9, 12 mo	HCC: promoting ART-adherence (self-report), immune function (viral load, CD4 cell count [blood tested in laboratory]) Other: reducing methamphetamine use (30-day recall + urinalysis), condomless anal sex (30-day recall)	210/210 included in primary analysis; 167, 173, 168, and 167 provided data at 3, 6, 9, and 12 mo, respectively	No significant intervention effects at any assessment on ART adherence, CD4, or viral load	Yes, moderation only (by information-motivation-behavior profiles found in prior study)	
Uusküla et al., 2018; two-arm RCT; Tallinn and Kohtla-Järve, Estonia; January-November 2013	PLHIV receiving routine clinical care at infectious disease clinics; N = 519	80%, problematic alcohol use (CAGE), 18% current injection drug use, 17% non-injection drug use, 15% opioid agonist therapy	Education and strengths-based counseling vs usual care; 12 mo	HCC: ART-adherence (3-day recall of missed doses), viral suppression (HIV RNA from clinical records; CD4 cell count also retrieved but not reported) Other: self-reported health status, ART beliefs, mortality	512/519 included in primary analysis; 93 lost to follow-up	Significant intervention effect at study endpoint on ART adherence, no effect on viral suppression	No	
Go et al., 2017; four-arm RCT; Thai Nguyen, Vietnam; July 2009-January 2011	PLHIV who inject drugs; N = 455	All participants had injected in the past six months; 54%, daily injection in past three months; 18% previously overdosed; 31% previously received drug treatment	Community intervention (stigma reduction) vs individual intervention (counseling addressing coping with stigma, social support, partner testing, disclosure, HIV knowledge, risk reduction, skill building) vs community + individual vs standard of care; 6, 12, 18, 24 mo	HCC: ART uptake (two self-reports, six mo recall), CD4 cell count (blood tested in laboratory) Other: mortality (attempted contact, tracing and report by family, verbal autopsy)	455/455 included in primary analysis; no other information provided	Significant intervention effect at study endpoint on ART uptake and mortality (CD4 was used to examine stratified effects)	Yes, mediation by self-reported overdose, depression symptoms, social support, visits to HIV providers, physician-reported opportunistic infections	
Wechsberg et al., 2018; two-arm RCT; Pretoria, South Africa; May 2012-September 2014	Black African women LHIV who use substances; N = 641	At baseline, 32% frequent heavy drinking (with 9 days of binge drinking in past 30 days) and daily drug use; 31% tested positive for marijuana, 18% opiates, 14% cocaine	Women’s Health CoOp Plus: HCT + two intervention sessions on substance use and sexual risk reduction, gender power, personalized action plans, case management; 6 mo, 12 mo	HCC: linkage to care, ART uptake (self-report), and viral load (dried blood spot testing) Other: condom use at last sex with partners, clients; substance use at last sex; frequent heavy drinking; fewer heavy drinking days; fewer drinking days; average number of drinks on typical drinking day; daily drug use; testing positive for marijuana, cocaine, opiates (urinalysis); gender-based violence (physical, sexual, emotional); condom negotiation, condom use while high, refusing sex without a condom (all self-report unless otherwise specified)	571 and 589/641 provided data at 6 and 12 mo, respectively	Significant intervention effect at study endpoint on viral suppression; no effects at study mid or endpoint on linkage and ART uptake	No	

RCT, randomized controlled trial; AUDIT, alcohol use disorders identification test; AUDIT-C, alcohol use disorders identification test-concise; DAST, drug abuse screening test; PLHIV, people living with HIV; ART, antiretroviral therapy; MSM, men who have sex with men; CAGE, cut, annoyed, guilty, eye; GAIN, global appraisal of individual needs; HIV, human immunodeficiency virus; CD4, cluster of differentiation 4; HCT, HIV counseling and testing; mo, month; f/u, follow-up

### Study characteristics

Ten articles reflected studies wherein substance-using populations were intentionally targeted, while one reflected a study with a population that incidentally used substances. Of the former, four studies involved the use of at least one of several substances, including alcohol, opioids, stimulants, and/or marijuana, among others; four involved injection drug use only; one involved methamphetamine use only; one involved alcohol use only. The population that used substances incidentally used alcohol, injection drugs, and non-injection drugs. Eight studies utilized a two-arm RCT design, two used a three-arm RCT design, and one used a four-arm RCT design. Geographic settings for studies included the United States (US; n = 5), South Africa (n = 1), Estonia (n = 1), Vietnam (n = 1), and Russia (n = 2); one study spanned Indonesia, Vietnam, and Ukraine. Moreover, differences between experimental arms were sometimes present, which we note below, and studies often used both objective (e.g., blood testing, clinical records) and subjective measures (e.g., self-report) to assess outcomes (Table 2).

**Table 2:** HIV care continuum outcomes with method of assessment for each study

Study	Linkage to Care	ART Uptake/Initiation	Retention in Care	ART Adherence	Immune Function (CD4 cell count)	Viral Load/Suppression
Attonito et al., 2019	Not examined	Not examined	Self-reported “service utilization” was measured using a validated item. “Are you currently receiving any of the following Treatment Services?” 52 services are listed (e.g., screening, recovery, case management, medical, after care, education, and peer-based recovery support services)	Self-reported percentage of time ART medications were taken as prescribed over the course of a week; “all” (100%), “most” (75%), “about half” (50%), “few” (25%), or “none” (0%) for each medication used; the mean adherence for all medications was calculated	Not examined	Self-report measure using a validated item. “Indicate your viral load the last time it was measured” with available responses: (1) undetectable, (2) 50–500, (3) 501–5000, (4) 5001–10,000, (5) 10,001–30,000, (6) 30,001 or more, (7) don’t know
Satre et al., 2019	Not examined	Not examined	Not examined	Self-report: “What is your best guess about how much of your prescribed HIV medications you have taken in the last month?” (dichotomized to ≥ 90% vs. < 90%).	Not examined	HIV viral control was abstracted from electronic health records
Samet et al., 2019	Medical chart review at 6- and 12-months	Medical chart review at 6- and 12-months	Medical chart review at 6- and 12-months	Not examined	At baseline and 12- month assessments, blood was collected for CD4 cell count testing.	Not examined
Samet et al., 2023	Not examined	ART initiation within 28 days of randomization, assessed via medical record	Defined as one or more visits to medical care in two consecutive 6-month periods, assessed via medical record	Not examined	Blood collected at baseline, 5, and 12-months, if blood draw was unsuccessful, the medical record was reviewed for closest available date	Blood collected at baseline, 5, and 12-months, if blood draw was unsuccessful, the medical record was reviewed for closest available date
Myers et al., 2018	Considered linked to care if participant had at least 1 documented nonurgent visit to a community medical provider within 30 days of their release from jail	Not examined	Considered consistently engaged in care during the follow-up year if participant had a nonurgent medical care visit between each of the follow-up visits (2, 6, and 12 months)	Not examined	Not examined	Abstracted viral load measures from both jail- and city-based laboratory databases
Metsch et al., 2016	Not examined	Current ART prescription measured using hospital medical record review	Self-reported HIV care visits assessed using a validated instrument (at least one visit to an HIV primary care provider in the past six months)	Self-report as the percentage of pills taken in the last 30 days	Not examined	Blood was drawn and tested at local laboratories
Miller et al., 2019	Not examined	Self-reported being on ART (or not)	Not examined	Not examined	Not examined	Blood samples were collected
Parsons et al., 2018	Not examined	Not examined	Not examined	Self-report, 14-day recall window was used for HIV medication adherence, using Timeline Follow-Back	Participants provided a blood sample collected onsite	Participants provided a blood sample collected onsite
Uusküla et al., 2018	Not examined	Not examined	Not examined	Self-reported adherence to ART (3-day recall measure)	Not examined	Medical data abstracted from clinical records
Go et al., 2017	Not examined	After each study visit, participants received a follow-up physical examination by the study physician where the physician asked about ART use in prior six months	Not examined	Not examined	Blood specimens collected	Not examined
Wechsberg et al., 2018	Self-reported linkage to care was assessed by the item “Have you been referred to a medical assessment?” Participants responded either 1 = Yes, went to medical assessment, 2 = Yes, but have not gone to medical assessment, or 3 = No.	Self-reported question, “Have you been prescribed any anti-HIV medications?”	Not examined	Not examined	Not examined	Whole dried blood spot samples were collected and prepared according to the recommended protocol from the World Health Organization

ART, antiretroviral therapy; CD4, cluster of differentiation 4

To provide a comprehensive overview of the findings from this systematic review, we first focus on describing each of the eleven studies in detail (organized by the primary substance used by the sample). For each study, we report the basic intervention components, sample, substance use-related characteristics, and significant intervention effects and other relevant findings, including outcomes that do not directly pertain to the HIV care continuum (e.g., reductions in substance use). Additional information for each study is described in Tables 1 and 2. At the end of this section we also describe overall findings of intervention effects on HIV care continuum outcomes. Results from the risk-of-bias assessment are described for each study and are summarized in Table 3. Overall risk-of-bias was deemed to be “low” across the studies, with the exception of three studies where there were “some concerns”.

**Table 3:** Overall Efficacy on HIV Care Continuum Outcomes and Risk of Bias Results

Author	Overall Efficacy on HIV Care Continuum Outcomes	Risk-of-Bias Assessment Domains						
		Randomization process	Deviations from the effect of assignment to intervention	Missing outcome data	Measurement of the outcome*	Selection of the reported result	Overall risk-of-bias judgment**	Noted concerns (if applicable)
Attonito et al., 2019	Significant intervention effects at study endpoint on ART adherence and viral suppression, no effect on retention (service utilization)	Low	Low	Low	Some concerns	Some concerns	Some concerns	Primary and secondary outcomes not pre-defined; Outcome measurements used self-report
Satre et al., 2019	No significant intervention effects at study mid or endpoints on ART adherence or HIV viral control	Low	Low	Low	Low	Low	Low	
Samet et al., 2019	Significant intervention effect at study midpoint on linkage to HIV care; no effects on ART uptake, retention, or CD4 at endpoint	Low	Low	Low	Low	Low	Low	
Samet et al., 2023	Significant intervention effect at study endpoint on viral suppression, ART uptake, and retention in care; no effect on CD4	Low	Low	Low	Low	Low	Low	
Myers et al., 2018	Significant intervention effects at study endpoint on linkage to and retention in care, no effect on viral load	Low	Low	Low	Low	Low	Low	
Metsch et al., 2016	Significant intervention effect on viral suppression, HIV care visits (retention), and ART use at study midpoint; no effect on ART adherence; no effects at study endpoint	Low	Low	Low	Low	Low	Low	
Miller et al., 2019	Significant intervention effects at study mid and endpoint on ART use and viral suppression	Low	Low	Low	Low	Low	Low	
Parsons et al., 2018	No significant intervention effects at any assessment on ART adherence, CD4, or viral load	Low	Low	Low	Low	Some concerns	Some concerns	Primary and secondary outcomes not pre-defined
Uusküla et al., 2018	Significant intervention effect at study endpoint on ART adherence, no effect on viral suppression	Low	Low	Low	Low	Low	Low	
Go et al., 2017	Significant intervention effect at study endpoint on ART uptake and mortality (CD4 was used to examine stratified effects)	Low	Low	Low	Low	Some concerns	Some concerns	Outcome analyzed in this paper was not a pre-defined primary or secondary outcome
Wechsberg et al., 2018	Significant intervention effect at study endpoint on viral suppression; no effects at study mid or endpoint on linkage and ART uptake	Low	Low	Low	Low	Low	Low	

*For the purpose of this systematic review, we focused on HIV care continuum outcomes in assessing the risk-of-bias for these outcomes; **Low risk of bias: The trial is judged to be at low risk of bias for all domains for this result; *Some concerns*: The trial is judged to raise some concerns in at least one domain for this result, but not to be at high risk of bias for any domain; *High risk of bias*: The trial is judged to be at high risk of bias in at least one domain for this result or the trial is judged to have some concerns for multiple domains in a way that substantially lowers confidence in the result.

### Multiple substances

Metsch et al. (2016) assessed the effect of a patient navigation intervention with and without financial incentives to promote ART uptake, ART adherence, and viral suppression among hospitalized PLHIV with elevated viral loads and past-year opioid, stimulant, or heavy alcohol use in 11 urban hospitals across the US [46]. An additional outcome involved outpatient care with an HIV specialist, which we considered retention in care [46]. At baseline, 59% of participants evidenced harmful/hazardous alcohol use; 97% had documented stimulant, opioid, or other drug use; 18% had injected drugs in the past year; and 70% evidenced severe substance use [46]. *HIV care continuum outcomes*. At six-month follow-up, more navigation-with-incentives participants were virally suppressed compared to control and navigation-only participants, and more navigation-with-incentives participants and navigation-only participants had attended HIV care visits and taken ART than control participants [46]. There was no intervention effect on viral suppression at 12 months [46]. *Other outcomes*. At six-month follow-up, more navigation-with-incentives and navigation-only participants received professional substance use disorder treatment than control participants [46]. There was no effect on other substance use outcomes at 12 months [46]. Risk-of-bias for this study was judged to be low (see Table 3).

Myers and colleagues (2018) assessed the effect of a patient navigation-enhanced HIV case management intervention to promote linkage to HIV care, retention in HIV care, and viral suppression, as well as reduce risky sex and drug use behavior among PLHIV reporting prior or current substance use and who were recently arrested and released from San Francisco County Jail [47]. At baseline, 94% of participants reported alcohol use in the 30 days prior to jail, 50% of whom reported alcohol use more than weekly before jail; 94% reported drug use in the 30 days prior to jail, 76% of whom reported weekly drug use before jail [47]. Methamphetamine use was the most reported drug used (63%; 40% used more than once per week), followed by crack-cocaine (57%; 37% used more than once per week) and heroin (30%; 13% used more than once per week) [47]. Thirty-three percent met criteria for alcohol abuse, 85% met criteria for substance abuse, with 8% meeting criteria for severe substance abuse [47]. Weekly drug use and using methamphetamines more than once per week in the 30 days before jail were significantly higher in the intervention group than in the control group [47]. *HIV care continuum outcomes*. Intervention participants were more likely to be linked to care within 30 days upon release and be retained in care over the subsequent 12 months [47]. Those who received substance dependence treatment in jail were more likely to be linked to care within 30 days upon release and be retained in care over the subsequent 12 months [47]. There was no effect on viral suppression [47]. *Other outcomes*. Intervention participants reported less risky sex at 12-month follow-up [47]. There was no intervention effect on alcohol or drug use behaviors [47]. Risk-of-bias for this study was judged to be low (see Table 3).

Satre et al. (2019) assessed the effect of motivational interviewing intervention (vs. emailed feedback vs. usual care) to reduce unhealthy alcohol use, alcohol problems, and drug use, and promote ART-adherence and viral load control among PLHIV with past-year unhealthy alcohol use recruited from an HIV primary care clinic in San Francisco [48]. At baseline, roughly 57% of participants were at high risk for alcohol use problems, and roughly 25% met criteria for alcohol dependence (no cross-arm differences) [48]. *HIV care continuum outcomes*. There were no effects on ART adherence or viral load control [48]. *Other outcomes*. There were declines in alcohol misuse within each arm but not between arms [48]. At the six-month follow-up, motivationally interviewed participants reported lower drug use/prescription drug misuse (excluding marijuana) than those in other arms [48]. Among participants reporting low importance of reducing alcohol use at baseline, those receiving motivational interviewing reported lower alcohol use at 12 months compared to those in other arms [48]. Risk-of-bias for this study was judged to be low (see Table 3).

Wechsberg et al. (2019) assessed the effect of an intervention (risk reduction and gender power) to reduce drug and alcohol use, gender-based violence, and sexual risk, and to promote linkage to HIV care, ART uptake, viral suppression, and sexual negotiation among Black women living with HIV who used at least one substance weekly for the past three months in Pretoria, South Africa [49]. At baseline, 32% of participants reported frequent heavy drinking (with nine days of binge drinking during the prior 30 days) and daily drug use; 31% tested positive for marijuana, 18% tested positive for opiates, and 14% tested positive for cocaine [49]. Also, at baseline, fewer intervention participants reported frequent heavy drinking (including days of binge drinking) than control participants, and fewer control participants reported daily drug use and tested positive for marijuana, opiates, and cocaine than intervention participants [49]. *HIV care continuum outcomes*. At 12-month follow-up, more intervention participants had undetectable viral load compared to control participants; there was no intervention effect on linkage to HIV care (among newly diagnosed, not-yet-linked participants) or ART uptake [49]. *Other outcomes*. At six-month follow-up, intervention participants reported decreases in alcohol use and physical and sexual intimate partner violence, increases in condom use, more frequent condom negotiation, and sex refusal without a condom with a partner in the past three months [49]. At the 12-month follow-up, intervention participants reported decreased emotional intimate partner violence [49]. Risk-of-bias for this study was judged to be low (see Table 3).

Uuskula and colleagues (2018) was the one study identified in this review that incidentally targeted a substance-using sample (most of the participants reported substance use). The study assessed the effect of an education and strengths-based counseling intervention on ART adherence and viral suppression among PLHIV receiving routine HIV clinical care from two infectious disease clinics in Tallinn and Kohtla-Järve, Estonia [50]. Roughly 80% of participants had problematic alcohol use, 18% reported current injection drug use, 17% reported current non-injection drug use, and 15% reported being currently on opioid agonist therapy (with no cross-arm differences) [50]. *HIV care continuum outcomes*. At the 12-month follow-up, ART adherence (≥ 95%) was marginally higher for those in the intervention group relative to the control group [50]. Among those with suboptimal ART adherence at baseline, more intervention participants reported optimal adherence at 12 months than control participants [50]. There was no effect on viral load [50]. *Other outcomes*. At 12 months, intervention participants viewed ART more favorably than control participants [50]. Risk-of-bias for this study was judged to be low (see Table 3).

### Injection drug use

Go and colleagues (2017) assessed the effect of a multilevel stigma-reduction intervention on ART uptake and survival among men who inject drugs (had injected in the past six months) in Thai Nguyen, Vietnam [51]. At baseline, 54% reported daily injection drug use in the past three months, 18% reported prior overdosing, and 31% had previously received drug treatment (no cross-arm differences) [51]. *HIV-care continuum outcomes*. Participants in the community plus individual-level intervention were more likely to initiate ART than standard-of-care participants [51]. *Other outcomes*. Among participants with a CD4 cell count < 200 cells/mm^3^ (ART-eligible threshold at the time) and not on ART at baseline, those in the community plus individual-level intervention had lower mortality than standard-of-care participants [51]. There were some concerns around risk-of-bias for this study (only in the domain of selection of the reported result), as the outcome analyzed in this paper was not a pre-defined primary or secondary outcome.

Miller et al. (2018) assessed the effect of an integrated harm reduction, systems navigation, and psychosocial counseling intervention to promote uptake and use of ART and medication-assisted drug treatment, and improve viral load suppression among PLHIV who inject drugs with elevated viral load in Kyiv, Ukraine; Thai Nguyen, Vietnam; and Jakarta, Indonesia [52]. *HIV care continuum outcomes*. At 12-month follow-up, uptake and use of ART and viral suppression were all higher for intervention participants than control participants [52]. *Other outcomes*. At 12-month follow-up, medication-assisted drug treatment was higher, and mortality was lower for intervention participants relative to control participants [52]. Risk-of-bias for this study was judged to be low (see Table 3).

Samet and colleagues (2019) assessed the effect of a peer-led strengths-based case management intervention to promote linkage to care, retention in care, and CD4 cell count testing at 12 months among PLHIV who inject drugs hospitalized at City Addiction Hospital in St. Petersburg, Russia [53]. Other outcomes included appropriate HIV care (prescribed ART or a second CD4 cell count if CD4 > 350 cells/µL) and self-reported hospitalizations at 12 months [53]. *HIV care continuum outcomes*. At 6-month follow-up, more intervention participants had been linked to HIV care than control participants, and at 12-month follow-up, more intervention participants had received appropriate HIV care than control participants [53]. There was no effect on CD4 cell count or retention in care [53]. *Other outcomes*. There was no effect on self-reported hospitalizations at 12 months [53]. Risk-of-bias for this study was judged to be low (see Table 3).

Samet et al. (2023) evaluated a multicomponent version [54] of the intervention tested in the RCT described above [53]. The 2023 RCT applied an intervention approach that combined peer-led strengths-based case management with rapid access to ART and receipt of naltrexone, a medication for opioid use disorder [54]. Like the prior study, the intervention was evaluated among PLHIV who inject drugs hospitalized at City Addiction Hospital in St. Petersburg, Russia. The primary outcome was viral suppression at the study endpoint (12-month follow-up). Secondary outcomes included ART initiation, change in CD4 cell count, and retention in HIV care. *HIV care continuum outcomes*. At study endpoint, a significantly larger proportion of participants in the intervention group relative to the control group had an undetectable viral load. Participants in the intervention group also had significantly higher odds of ART initiation and retention in HIV care. No changes were observed for CD4 cell count across follow-up [54]. *Other outcomes.* Clinically meaningful differences in opioid abstinence were observed between the two study arms at 6 and 12 months, but these differences were not statistically significant [54]. Risk-of-bias for this study was judged to be low (see Table 3).

### Methamphetamines

Parsons et al. (2018) assessed the effect of motivational interviewing plus cognitive behavioral therapy intervention on reducing methamphetamine use, condomless anal sex, ART adherence, viral suppression, and immune function (increased CD4 cell count) among cisgender sexual minority men living with HIV who use methamphetamines with suboptimal ART-adherence in New York City [55]. At baseline, participants reported methamphetamine use an average of roughly six days in the past month (no cross-arm differences) [55]. *HIV care continuum outcomes*. At 3-, 6-, 9-, and 12-month follow-ups, intervention and control participants evidenced increased ART adherence and CD4 cell count (and lower viral load) compared to baseline, but there was no difference between arms [55]. Moderation by Information-Motivation-Behavior class (identified in a prior study) emerged: among participants in the “global barriers” class (i.e., those with global barriers to changing their methamphetamine use and medication adherence), those in the intervention had a greater improvement in ART adherence than those in the control condition [55]. *Other outcomes*. At 3-, 6-, 9-, and 12-month follow-ups, intervention and control participants evidenced reduced substance use and condomless anal sex, but there was no difference between arms [55]. There were some concerns around risk-of-bias for this study (only in the domain of selection of the reported result), as the study did not assess pre-defined primary or secondary outcomes.

### Alcohol

Attonito and colleagues (2020) assessed the effect of a holistic health intervention to improve ART adherence and viral load among PLHIV reporting harmful/hazardous drinking recruited from substance abuse treatment facilities and HIV care and service organizations in Miami, Florida [56]. An additional outcome was HIV service utilization, which we considered retention in care [56]. Forty-six percent of participants reported prior treatment for alcohol use [56]. *HIV care continuum outcomes*. At six-month follow-up, intervention participants were more likely to report optimal ART adherence (≥ 95%) and undetectable viral load. HIV service utilization improved for both arms, but there was no difference between arms [56]. *Other outcomes*. At six-month follow-up, intervention participants were more likely to report greater social support than control participants [56]. There were some concerns around risk-of-bias for this study (in the domains of measurement of the outcome and selection of the reported result), as the HIV care outcomes analyzed in this paper relied solely on self-report, and the study did have pre-defined primary or secondary outcomes.

### Intervention effects on HIV care continuum outcomes

We examined the overall efficacy of the different studies in promoting HIV care continuum outcomes. All of the studies assessed at least two different HIV care outcomes (see Table 2). Of the eleven studies reviewed, only two found statistically significant intervention effects on all HIV care outcomes that were examined [51, 52]. These two studies were among the studies included in this review that focused on people who inject drugs and were conducted outside the US. The interventions in these two studies applied a multi-level approach. Specifically, Miller and colleagues applied harm reduction, systems navigation and psychosocial counseling to promote HIV care outcomes [52]; whereas Go and colleagues compared community- vs. -individual-level interventions [51]. Two studies did not find any significant effects on the HIV care outcomes that were examined [48, 55]. The interventions in these studies were applied solely at the individual level, and were the only studies in this review that relied mainly on motivational interviewing techniques as the intervention approach. The other seven studies found mixed effects, in that significant differences were observed between intervention and comparison arms for one or more HIV care outcomes, but not for others (e.g., for linkage to care but not CD4 cell count). The most commonly examined HIV care outcome was viral suppression, measured in nine (82%) of the eleven studies. Out of these nine studies, four (44%) found significant intervention effects on viral suppression at study endpoint [49, 52, 54, 56]. However, there were some concerns regarding risk-of-bias in one of the three studies [56].

## Discussion

This systematic review identified eleven behavioral HIV care continuum intervention studies using an RCT design. These studies were published from 2011 to 2023 and either intentionally or incidentally targeted substance-using populations living with HIV. Populations using single or multiple substances, spanning alcohol, injection drugs, and non-injection drugs were sampled in low-, middle-, and high-resource countries and ranged in size from N = 210 to N = 1308. For all studies, the measures used to assess active substance use were valid or well-established approaches, including use of validated screening tools (e.g., AUDIT or CAGE), confirmation of injection drug use through evidence of injection markings, or admission in a hospital for substance use disorder, or assessment of specific substance use (e.g., methamphetamines) in a specified timeframe (e.g., past 30 days). Most of the behavioral interventions targeted individuals (e.g., skill-building, reducing substance use), though some also targeted healthcare systems or communities (e.g., patient navigation, case management, stigma mitigation). Interventions sought to address multiple HIV care continuum outcomes, including linkage to, receipt of, and retention in care; ART uptake and adherence; immune function; and viral suppression, with varying efficacy. Due to the diversity of substances used across the eleven studies, as well as the diversity in intervention approaches across the board and within each substance use domain, we were not able to identify a pattern of findings based on the substance of focus (e.g., alcohol vs. injection drug use). Therefore, for the discussion, we focus on interpreting findings based on the HIV care outcomes that were examined.

Viral load was the most commonly targeted HIV care continuum outcome (9/11 studies), and this is unsurprising, as viral suppression is the primary goal of HIV care continuum progression and motivated the transition to the TasP era [25]. Viral suppression was achieved at study endpoints in four interventions (with heavy alcohol-using, injection drug-using, and polysubstance-using samples), and at the midpoint only in one intervention (with a polysubstance-using sample), which underscores the extent to which any substance use may compromise HIV care efforts. However, the fact that intervention efficacy was demonstrated nonetheless shows promise for supporting viral suppression among substance-using populations. The diversity of substances examined across the handful of efficacious studies (in addition to other factors) precludes making any overall deductions or conclusions about which substances may be most amenable to intervention to support viral suppression. Further substance-specific intervention research is needed, including interventions targeting specific substances (e.g., stimulants) that are known to independently increase viral load [10].

ART uptake and ART adherence were the second-most commonly targeted HIV care continuum outcomes (7/11 studies each), with greater ART initiation by endpoints in five interventions, and with greater ART adherence being achieved by endpoints in three interventions. ART uptake and adherence are necessary to achieve viral suppression and undetectability (and improve overall immune function) but remain vulnerable to the prioritization and effects of substance use [13]. Alcohol was the primary substance used in the studies that found significant intervention effects on ART adherence, suggesting that ART adherence even in the context of heavy alcohol use may be amenable to intervention, but this requires further investigation. ART adherence in the context of other substance use, and ART adherence as a mediator between intervention and viral suppression in trials are also areas for future research.

Retention in care was a less commonly targeted outcome (5/11 studies). Three studies explicitly identified retention in care as an intervention target, and two identified HIV service utilization and attending HIV care visits as intervention targets. Greater retention in care was achieved at the endpoint of two interventions and at the midpoint of another. Retention in care remains an important stage of the HIV care continuum leading to viral suppression, and clear disparities in retention in care have been demonstrated for people who inject drugs (or have a history of such) relative to those who do not inject or have not injected drugs [57]. Interventions targeting retention in care among people who inject drugs or have a history of injection drug use may be warranted.

Linkage to care was also less commonly targeted (3/10 studies), possibly due to the fact that universal test-and-treat strategies were being or beginning to be implemented during our pre-specified time period for article inclusion (2011 onward), or due to linkage to care being framed similarly to retention in care (healthcare system vs individual responsibility) [58–61]. However, because no main intervention effects on linkage to care were maintained at any study’s endpoints, linkage to care may be an appropriate intervention target for future trials. Relatedly, none of the identified interventions targeted HIV diagnosis or status awareness as a precursor to linkage to care and entering into the HIV care continuum. Substance use can lead to delays and even intentional avoidance of HIV testing and delayed linkage to HIV care among PLHIV [13]. Like linkage to care, HIV testing and status awareness may be inadvertently neglected in intervention efforts with substance-using populations living with HIV. Testing and status awareness may need to be reconsidered in future intervention development and testing endeavors.

In this review, we identified an important similarity among interventions shown to be efficacious in affecting HIV care outcomes at study endpoints. In addition to targeting individual-level factors, these interventions expanded beyond individuals. They targeted broader concerns – such as interpersonal-level (social support), healthcare system-level (patient navigation, case management), and community-level factors (stigma mitigation) – to varying extents. Though substance use is an individual behavior and some HIV care continuum stages involve individual behaviors (e.g., ART adherence), they are not enacted in a vacuum. A range of socioecological factors shape substance use and HIV prevention and care efforts [62–65]. Interventions targeting multiple, especially higher-order socioecological levels may be more effective for supporting substance-using populations living with HIV.

Mediation and/or moderation analyses were infrequently conducted in the identified studies, potentially masking additional findings that may prove statistically, clinically, or behaviorally significant. Prior research has demonstrated the value of revisiting trial data to examine mediators and moderators of findings, including non-significant findings [66]. Primary outcome analysis is often restricted to main effects averaged over all participants, and reanalyzing data from those trials (whether originally efficacious or not) by including mediators and/or moderators in analytic models may reveal scientifically and practically important findings. For example, mediation and moderation analysis of trial data may yield significant findings that were initially undetected in the primary outcome analysis, including new intervention-to-outcome mechanistic pathways, or previously unidentified subgroups for whom the intervention demonstrated greater or lesser efficacy [66]. Notably, several studies in this review reported significant intervention effects on non-HIV care continuum outcomes (e.g., reduced substance use, condomless sex, gender-based violence, increased social support). These other outcomes may act as mediators or moderators of the intervention effect on HIV care continuum outcomes. They should be explored in future research, whether through secondary analyses of data from these completed trials or through planned analyses of new intervention trials.

We did not identify any PrEP-related studies that met our inclusion criteria in our search. However, screening and examining PrEP-related articles for inclusion in this review suggested that there is growing intervention attention to promoting engagement in the PrEP care cascade among people who use drugs, yielding four protocol papers for intervention studies currently underway for this population [67–70]. Moreover, a systematic review of the PrEP care cascade among people who inject drugs [29] described a feasibility and acceptability study for an intervention to increase PrEP adherence among people who inject drugs [71]. Prompt and rigorous evaluation of these interventions will be useful for understanding how to support people who use drugs and alcohol progress across the PrEP care cascade.

Evidence-based intervention approaches to treat substance use disorder include medications for opioid use disorder and contingency management [72, 73]. These approaches have also been demonstrated to improve HIV care-related outcomes [74, 75]. Only two studies included in this review applied these types of substance use treatment approaches. Specifically, Metsch et al. (2016) evaluated continency management, or the opportunity to receive financial incentives when specific target behaviors were achieved, including for example submitting drug- and alcohol-negative urine specimens [46]. Also, Samet et al. (2023) provided extended-release naltrexone to participants in the intervention [54]. Both interventions yielded significant effects on viral suppression, one at study midpoint [46] and the other at endpoint [54]. More research should be conducted in the future to continue to evaluate the impact of substance use disorder treatment interventions (e.g., medications) on HIV care continuum outcomes among people living with HIV, either alone or in combination with other behavioral HIV care intervention approaches (similar to Samet et al. [2023] which included peer-led strengths-based case management, rapid ART, and naltrexone [54]). Indeed, behavioral interventions alone may not lead to improving HIV care outcomes specifically among people with HIV with opioid use disorder or alcohol use disorder, and the combined use of behavioral approaches along with medications are likely to be most effective.

It is important to note that the search terms (i.e., “HIV intervention”) and inclusion criteria for the review (i.e., study focused on people at risk for or living with HIV) may have failed to locate RCT intervention studies that were not primarily focused on assessing an HIV care intervention specifically, but may have still included people who use drugs and assessed HIV care outcomes. For instance, there may be previous RCT studies that primarily evaluated substance use treatment intervention and assessed HIV care outcomes. Such studies may not have been picked up in this review. Thus, a future systematic review that includes an expanded focus on both substance use and HIV treatment interventions is warranted. Of note, there was a systematic review that was published in 2021 on medications for opioid use disorder, and their effect on infectious disease outcomes, including HIV care outcomes [22]. The present study fills a gap in the literature by focusing specifically on behavioral HIV care interventions.

There are several limitations to this review. Since our search included terms related to drug, alcohol, or substance use, we may have failed to locate other studies in which populations with incidental substance use were sampled, as such studies may not have been indexed using those terms at the time of publication or may have reflected interventions that did not target substance-using populations. Second, several factors prevented easy between-study comparisons and limited generalizability of findings: the diversity of substances and drug use behaviors examined across studies, the variations in substance use measurement (for studies that did involve the same substance or behavior), the range of countries (low and middle income versus high income) and contexts (urban cities; hospitals, jails) where studies were conducted, and the different HIV treatment policies in place at the time studies were conducted. Third, several studies examined self-reported rather than objectively or biologically measured outcomes, potentially resulting in reporting bias. Finally, we focused only on intervention outcomes published since 2011. A review inclusive of studies published prior to the TasP era may have painted a different picture of the landscape of HIV care continuum outcomes among people who use substances.

## Conclusions

Globally, substance use remains a prevalent health behavior and a barrier to HIV care continuum progression among PLHIV. Several behavioral HIV care interventions have been conducted with people who use substances in the TasP era, and there were mixed findings with regards to effects on various HIV care continuum outcomes. However, there is much more to learn regarding intervention effects among people who use substances and are living with HIV, given the relatively low number of studies identified here, the diversity of substances examined, the diversity of intervention approaches applied, and the potential for nuanced effects (e.g., mediated and moderated effects). Additional intervention research specific to the use of certain substances across diverse samples, as well as research involving the re-examination of data from previous trials to tease out mediated and moderated intervention effects, can illuminate more clearly how to support PLHIV who use substances.

## Data Availability

Our specific collection of data that we extracted from the studies are not currently available; however, as this is a systematic review, all data included in the review are available in the original articles from which the data were extracted

## List of abbreviations

PLHIV: people living with HIV
TasP: treatment as prevention
PrEP: pre-exposure prophylaxis
